# Broad-spectrum ubiquitin/ubiquitin-like deconjugation activity of the rhizobial effector NopD from *Bradyrhizobium* (sp. XS1150)

**DOI:** 10.1038/s42003-024-06344-w

**Published:** 2024-05-27

**Authors:** Ying Li, Jordi Perez-Gil, L. Maria Lois, Nathalia Varejão, David Reverter

**Affiliations:** 1 Institut de Biotecnologia i de Biomedicina and Dept. de Bioquímica i Biologia Molecular, Universitat Autònoma de Barcelona, 08193 Bellaterra Barcelona, Spain; 2 Center for Research in Agricultural Genomics-CRAG, Edifici CRAG-Campus UAB, 08193 Bellaterra Barcelona, Spain; 3 Consejo Superior de Investigaciones Científicas (CSIC), Barcelona, Spain; 4 Institució Catalana de Recerca i Estudis Avançats (ICREA), Barcelona, Spain; 5 Present Address: Qingdao University, 266071 Qingdao, China; 6 Present Address: ARC Centre of Excellence in Synthetic Biology and Centre for Agriculture and the Bioeconomy, Queensland University of Technology, Brisbane, QLD 4000 Australia

**Keywords:** X-ray crystallography, Proteases, Sumoylation

## Abstract

The post-translational modification of proteins by ubiquitin-like modifiers (UbLs), such as SUMO, ubiquitin, and Nedd8, regulates a vast array of cellular processes. Dedicated UbL deconjugating proteases families reverse these modifications. During bacterial infection, effector proteins, including deconjugating proteases, are released to disrupt host cell defenses and promote bacterial survival. NopD, an effector protein from rhizobia involved in legume nodule symbiosis, exhibits deSUMOylation activity and, unexpectedly, also deubiquitination and deNeddylation activities. Here, we present two crystal structures of *Bradyrhizobium* (sp. XS1150) NopD complexed with either *Arabidopsis* SUMO2 or ubiquitin at 1.50 Å and 1.94 Å resolution, respectively. Despite their low sequence similarity, SUMO and ubiquitin bind to a similar NopD interface, employing a unique loop insertion in the NopD sequence. In vitro binding and activity assays reveal specific residues that distinguish between deubiquitination and deSUMOylation. These unique multifaceted deconjugating activities against SUMO, ubiquitin, and Nedd8 exemplify an optimized bacterial protease that disrupts distinct UbL post-translational modifications during host cell infection.

## Abstract (web summary)

The unique structural features of the NopD catalytic domain, an effector protease from symbiotic bacteria rhizobia, grant a surprising versatility to remove various regulatory tags (SUMO, ubiquitin, Nedd8) from host plant proteins.

## Introduction

Bacterial infection and colonization in eukaryotic host cells requires special mechanisms to suppress host defense systems and ensure pathogen or symbiont survival. A common strategy by infectious bacteria consist on the injection of a plethora of effector proteins into the eukaryotic cell host by complex multiprotein structures, such as the needle-like Type III secretion system (T3SS), which spans the inner and outer bacterial membranes and is highly conserved among bacterial pathogens^1^. T3 protein effectors are injected by pathogenic bacteria into eukaryotic host cells to hijack the host defense systems. In plants, in addition to pathogenicity, T3SS has also been identified in rhizobia, a symbiotic bacteria that reside in legumes and can fix the atmospheric nitrogen to ammonia by the formation of nodules in the roots of legume plants^2,3^. The effector proteins of the rhizobia T3SS are called Nops (Nodulation Outer Protein), and around 20 to 30 T3 effector proteins have been identified into the legumes host cells, such as NopL^4^, NopE1/E2^5^, NopM^6^, NopT^7^ and NopP^8^. Signaling kinase cascades or the eukaryotic UPS (Ubiquitin-Proteasome system) are pathways often targeted in the host by T3 bacterial effectors to allow infection^9^.

Ubiquitin can regulate a large number of cellular processes in the eukaryotic cells by the formation of distinct polymeric chains, including protein half-life, localization, endocytosis, autophagy, and many more major functions^10^. Ubiquitin is ubiquitous and highly conserved in eukaryotes, including animals, yeast, and plants. Additionally, Ubiquitin-like modifiers (Ubl), such as SUMO, Nedd8 or ISG15, can also regulate many cellular pathways by covalent modification of protein targets^11^. In general, UbL modification (ubiquitin, SUMO or Nedd8) is conducted by an enzymatic cascade orchestrated by an E1 activating enzyme, an E2 conjugating enzyme, and E3 ligases^12^. Ubiquitin or SUMO modification can be reversed by the action of several dedicated families of proteases called deubiquitinases (DUBs) and deSUMOylases (SENP/ULP). Humans contain around 100 DUBs grouped in 7 different families based on their structure and action mechanisms^13^. DUBs are mostly specific for ubiquitin and its polymeric chains^14^, although in some cases they can cleave off other UbLs from protein targets, such as ISG15 (USP18) or SUMO (USPL1). The deSUMOylase CE clan of cysteine proteases in humans consists of six SUMO-specific proteases (SENP1-3 & 5-7) and one NEDD8-specific protease (NEDP1/SENP8)^15^.

Also, a number CE family enzymes are encoded in pathogenic bacteria and in bacteria that reside inside eukaryotic cells (plant symbiont)^2,3^. Since no Ubl-type modifier systems exist in these bacteria, they are injected as effectors to manipulate the eukaryotic host signaling pathways. Some of these CE family effectors include the SdeA from Legionella^16^, ChlaDUB1 from *Chlamydia*^17^, LegCE from *Legionella*^18^, SseL from *Salmonella*^19^, XopD from *Xanthomonas*^20^, ElaD from *E. coli*^21^, OtDUB from *Orientia*^22^, RickCE from *Ricksettia*^18^ and ShiCE from *Shigella*^18^. Interestingly, most bacterial CE proteases are specific for ubiquitin instead of SUMO, despite their structural similarity with the SENP/ULP family^18^. In some cases they even possess an unusual Ser/Thr acetyltransferase activity, such as YopJ from *Yersinia*^23,24^. Structural comparisons between bacterial protease effectors from different organisms indicate a remarkable degree of versatility within the CE catalytic domain, enabling them to acquire specialized functions^18^. This is exemplified by the ability of some effectors to cleave diverse polyubiquitin chain linkages.

Recently in rhizobia a novel T3 effector was identified belonging to the CE protease clan with specific activity against plant SUMOs^25^. NopD, from *Bradyrhizobium sp*. XS1115, contains a C-terminal CE protease domain with sequence similarity to XopD, a T3 effector from the plant pathogen *Xanthomonas campestris*^26^. Remarkably, *Xanthomonas* XopD possesses a unique dual enzymatic function, exhibiting both deSUMOylation and deubiquitinating activities. This versatility stems from its ability to form two distinct binding interfaces, allowing it to cleave either SUMO or ubiquitin^18^. This dual specificity for SUMO and ubiquitin is a rare feature among eukaryotic DUBs and SENP/ULP family members.

A recent characterization of rhizobia NopD indicate that the CE protease activity is required for cell death induction in tobacco and it can reduce the size of the nodule formation in a model legume plant *Tephrosia vogelii*^25^. Also, several NopD-like effector proteins have been identified in different rhizobia species possessing a CE catalytic protease domain similar to XopD^27^, such as Bel2-5 in *Bradyrhizobium elkanii* (USDA61 strain*)*, which seems to have a role in root nodule formation^28^, or MA20_12780 from *Bradyrhizobium japonicum* (Is-34), also suggested to act as a virulence factor in nodulation^29^. In all instances, the deconjugation activity of T3 NopD-like effectors have a major role during rhizobia infection and nodule formation in legume roots.

In this work, we demonstrate that *Bradyrhizobium* (sp. XS1150)‘s T3 effector protein, NopD, exhibits not only deSUMOylation activity but also K48-specific deubiquitinating and deNeddylating activities. This feature of having multipotent deconjugation activity within a single catalytic domain for SUMO, ubiquitin, and Nedd8 is uncommon among bacterial effector proteins. We have elucidated the molecular mechanism underlying this deconjugation activity by solving the crystal structures of NopD in complex with either Arabidopsis SUMO2 or ubiquitin at 1.5 or 1.9 Å resolution, respectively. Notably, despite the low conservation on the surfaces of SUMO and ubiquitin, NopD binds to both UbLs using a similar interface. Structural analysis and in vitro assays reveal the specific residues within the NopD interface that are responsible for its multiple SUMO, ubiquitin, and Nedd8 activities.

## Results

### NopD has an unusual multiple activity for SUMO, ubiquitin and Nedd8

Structural predictions indicate that the 1017 residue full-length protein of the *Bradyrhizobium* NopD symbiotic effector consists of a long N-terminal extension, which based on the Alphafold-2 model is formed by a combination of disordered regions and small helical globular domains, followed by a globular C-terminal protease domain with homology with the CE clan of cysteine proteases. This C-terminal catalytic domain was reported to possess deconjugating activity for plant SUMOs^25^, displaying the highest homology with XopD, a bacterial plant effector protease from *Xanthomonas campestris* (22.3% sequence identity for 184 residues)^26^. The catalytic domain of NopD also displays low homology with the eukaryotic ULP/SENP deSUMOylase family. So, based on sequence alignments with members of the ULP/SENP family (Supplementary Fig. 1), we generated a C-terminal catalytic domain construct of NopD from Gly832 to Asn1017.

**Fig. 1:**
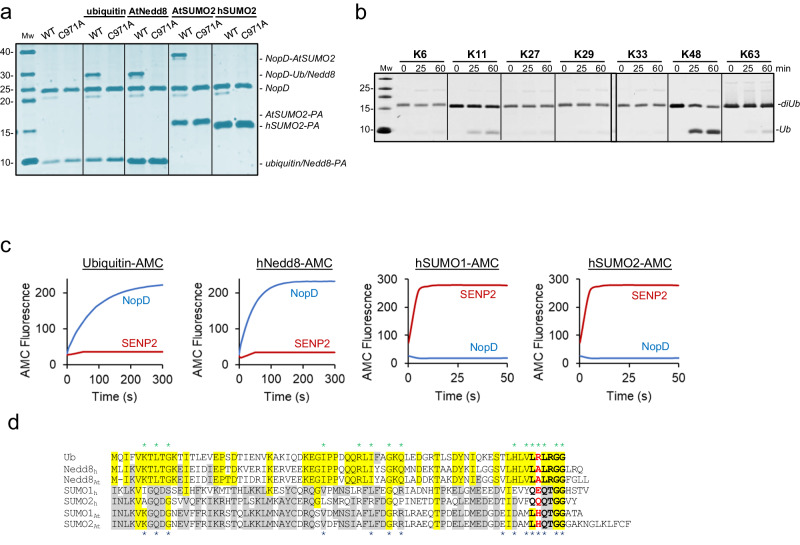
Specificity of NopD for *Arabidopsis* SUMO, ubiquitin and Nedd8. **a** SDS-PAGE of the binding between the NopD wild-type and active site mutant C971A against ubiquitin-PA, *Arabidopsis* Nedd8-PA, *Arabidopsis* SUMO2-PA and human SUMO2-PA activity-based probes for 2 h. **b** SDS-PAGE of the di-ubiquitin linkage specificity analysis for NopD over time. The concentration of NopD and diUb are 600 nM and 3 μM, respectively. **c** Time-course plots of fluorescent AMC-based substrates (100 nM) of ubiquitin, human Nedd8, human SUMO1 and SUMO2 substrates with the catalytic domains of *rhizobia* NopD (25 nM) and human SENP2 (25 nM) as a control. Reactions were conducted in triplicate and the average curve is displayed. **d** Sequence alignment of ubiquitin, human and *Arabidopsis* Nedd8 and, human and *Arabidopsis* SUMO1 and SUMO2. Yellow and grey background depict sequence conservation between ubiquitin and Nedd8, and between SUMO isoforms, respectively. The C-terminal tails before the diGly motif are marked in black bold letters. Ubiquitin Arg72 position in marked as red bold letter. Asterisks indicate binding residues of NopD with either *Arabidopsis* SUMO2 (blue) or ubiquitin (green), respectively.

Unexpectedly, our initial in vitro covalent modification assays with activity-based probes (ubiquitin-PA or UbL-PA) indicate that the catalytic domain of *rhizobium* NopD displays a preference for plant SUMO, ubiquitin, and plant Nedd8 (Fig. 1a). NopD binds *Arabidopsis* SUMO2-PA, ubiquitin-PA and Nedd8-PA probes, in contrast to the lack of binding to human SUMO2-PA. The specificity of the interaction was demonstrated by the absence of crosslinked adducts when catalytic inactive active site cysteine to alanine mutants (C971A) were analyzed (Fig. 1a).

Based on our initial binding to ubiquitin, we next checked the *rhizobium* NopD preference for the different types of poly-ubiquitin chains by using a di-ubiquitin chain assay kit (Fig. 1b). The symbiotic *rhizobia* NopD displays a preference for K48 ubiquitin linkages in vitro, and to a lesser extent for K11 linkages (Fig. 1b), in contrast to the CE clan of bacterial pathogens in humans that usually display deubiquitinating specificity for K63-linked chains^18,19,21^.

Moreover, in addition to plant SUMO and ubiquitin, *rhizobia* NopD also exhibits a strong deconjugating activity for Nedd8 (Fig. 1). NopD displays deconjugating activity against Nedd8 and ubiquitin using fluorescent-AMC substrates, but it is not active against human SUMO1 or SUMO2. In contrast to the human deSUMOylase SENP2, which is only active for human SUMO1 and SUMO2 (Fig. 1c). Sequence alignment shows a low homology between human or plant SUMOs with either ubiquitin or Nedd8, being the latter two quite similar (Fig. 1d). The existence of a deconjugating protease, such as *rhizobia* NopD, which is simultaneously active for ubiquitin, Nedd8 and SUMO is quite uncommon, and represents a paradigmatic example of an optimized catalytic domain containing multiple UbL deconjugating activities during host infection.

### Overall complex structures of NopD with *Arabidopsis* SUMO2 and ubiquitin

To form complexes of NopD with either *Arabidopsis* SUMO2 and ubiquitin, the C-terminal carboxylate groups of SUMO and ubiquitin were chemically modified with a highly reactive and stable C-terminal alkyne (SUMO2-PA or ubiquitin-PA) after a reaction with propargylamine^30–32^. The C-terminal carboxylate group of SUMO2-PA or ubiquitin-PA can thus be crosslinked with the active site cysteine of NopD (Cys971) to form a stable covalent product complex after incubating NopD with either SUMO2-PA or ubiquitin-PA. In an initial screening, a few diffraction quality crystals of NopD-SUMO2 or NopD-ubiquitin were grown in conditions containing 0.1 M imidazole (pH 7.0), 50% MPD or 0.1 M imidazole (pH 8.0), 10% PEG8000, respectively. Molecular replacement with human ubiquitin (PDB 1UBQ), *Arabidopsis* SUMO2 and NopD Alphafold-2 models assisted to determine the complex structures. Two complexes per asymmetric unit were found in the NopD-SUMO2 crystals, which belonged to the P2_1_2_1_2_1_ space group and diffracted to a 1.50 Å resolution; and one complex per asymmetric unit in the NopD-ubiquitin crystals, which belonged to the P4_3_ space group and diffracted beyond 1.94 Å resolution (Table 1).

**Table 1:** Crystallographic statistics of NopD-AtSUMO2 and NopD-Ubiquitin crystal structures

	*NopD-AtSUMO2*	*NopD-ubiquitin*
Data collection		
Space group	P2_1_2_1_2_1_	P4_3_
Unit cell parameters (Å)	80.85, 86.83, 90.49	86.50, 86.50, 46.67
Wavelength (nm)	0.9791	0.9792
Resolution range (Å)	49.52–1.50	43.25–1.94
R_merge_	0.07 (0.83)	0.07 (1.42)
R_pim_	0.03 (0.48)	0.03 (0.60)
(I/σ(I))	14.2 (1.8)	13.3 (1.2)
Completeness (%)	92.7 (48.9)	94.2 (55.0)
Multiplicity	6.1 (3.7)	6.8 (6.6)
CC (1/2)	0.99 (0.49)	0.99 (0.51)
Structure refinement		
Resolution range (Å)	49.52–1.50	43.25–1.94
No. of unique reflections	76875	22770
R_work_ / R_free_ (%)	17.84/20.36	17.16/19.94
No. of atoms		
Protein	8363	2055
Water molecules	312	62
Overall B factors (Å^2^)	31.02	59.74
Rms deviations		
Bonds (Å)	0.015	0.007
Angles (°)	1.140	0.839
Ramachandran favored (%)	99.22	98.41
Ramachandran allowed (%)	0.59	1.19
Ramachandran outliers (%)	0.2	0.4
PDB code	8OI3	8RQI

**Fig. 2:**
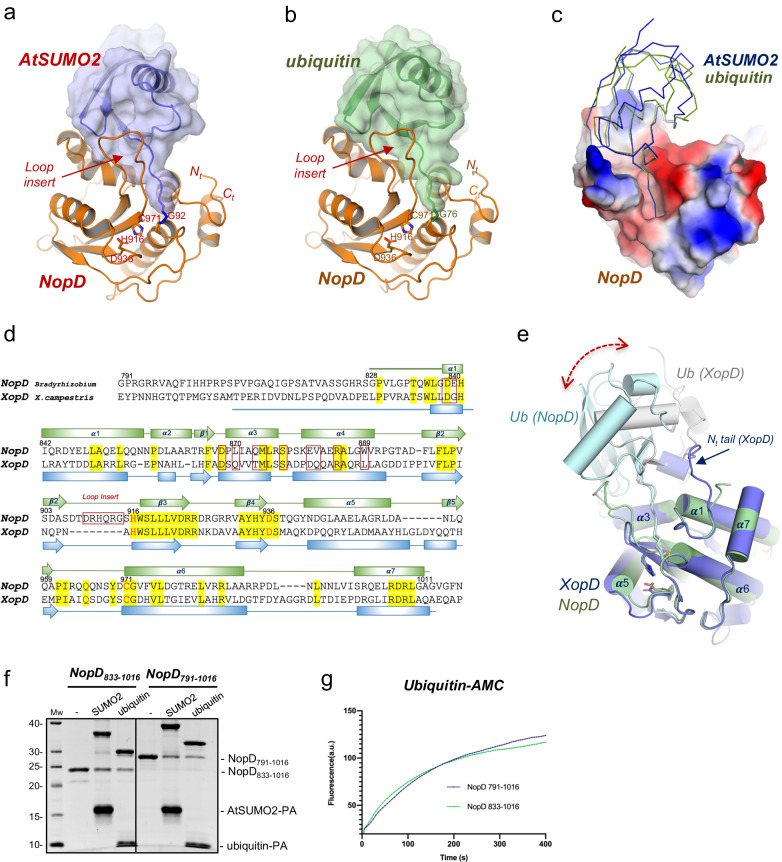
Crystal structure of the complexes between NopD and AtSUMO2 and ubiquitin. **a** Cartoon representation of the NopD catalytic domain with *Arabidopsis* AtSUMO2. **b** Cartoon representation of the NopD catalytic domain with ubiquitin. Catalytic triad residues are labeled and shown in stick representation. The N- and C-terminal are labeled. Loop insert in NopD is labeled and marked. **c** Electrostatic potential surface representation of NopD in complex with overlapped ribbon structures of *Arabidopsis* SUMO2 (blue) and ubiquitin (green). **d** Structural alignment of sequences corresponding to the catalytic domains for NopD and Xanthomonas campestris XopD. Red squares indicate interface residues to NopD. Catalytic triad residues are shown in red. Secondary structure cartoon is depicted above for NopD (green), or below for XopD (blue). **e** Superposition of the NopD-ubiquitin complex (green) with the Xanthomonas XopD-Ubiquitin (PDB: 5JP3) complex (blue). Double headed discontinued blue arrow indicates the displacement of ubiquitin between NopD (grey) and XopD (light blue) complexes. **f** SDS-PAGE of binding of NopD with a longer N-terminal extension against AtSUMO2-PA and ubiquitin-PA suicide probes for 2 h. Bands of NopD crosslink, NopD_791-1016_, NopD_833-1016_, AtSUMO2-PA, ubiquitin-PA are labeled. **g** Time-course of ubiquitin-AMC hydrolysis for NopD_833-1016_ and NopD_791-1016_ constructs. Ub-AMC (100 nM) was incubated with NopD (5 nM) constructs over time. Reactions were conducted in triplicate and the average curve is displayed.

The catalytic domain of NopD displays the canonical fold of CE protease clan and revealed a continuous chain from Pro829 to Ala1011 in complex with SUMO2, and from Gly828 to Leu1009 in complex with ubiquitin (Fig. 2a, b). The final electron density maps show the covalent bond formed between the SUMO or ubiquitin C-terminal glycine and the NopD active site Cys971, confirming the specificity of the catalytic reaction between NopD with SUMO2-PA or ubiquitin-PA substrates (Supplementary Fig. 2). NopD interacts with a similar interface with SUMO and ubiquitin, despite their low homology in the contact surface residues (Fig. 2c and Supplementary Fig. 4).

The structural overlapping with the AlphaFold-2 model of NopD (unbound) displayed mainchain rmsd values (root mean square deviation) of 0.93 Å and 0.90 Å in complex with SUMO and ubiquitin, respectively (Supplementary Fig. 3). The active site catalytic triad seems already formed in the absence of SUMO or ubiquitin substrate, as inferred by the distances between the catalytic triad residues (Cys971, His916 and Asp936) between our complex structures with the AlphaFold-2 model, suggesting that NopD might be active in the absence of a UbL substrate, as initially observed in human SENP2^33^, and does not require any substrate-induced activation mechanism.

To rule out a role for the N-terminal extension of NopD in ubiquitin binding, as observed in the *Xanthomonas* XopD-ubiquitin structure^18^, we produced a NopD catalytic domain with an extended N-terminal region starting at Gly791 (Fig. 2d). Our in vitro assays indicate that the NopD N-terminal extension does not significantly affect its binding properties or catalytic activities, as evidenced by similar results in both NopD constructs (Fig. 2f, g). Thus, in the NopD complex, a different interface is formed compared to XopD, as indicated by a ubiquitin displacement of approximately 5–6 Å when superimposing both complexes (Fig. 2e). Interestingly, the NopD interface with ubiquitin and AtSUMO2 remains similar, despite the low sequential homology in their surface contact residues (Fig. 1d and Supplementary Fig. 4).

An important unique characteristic in the NopD structure is the presence of a “Loop insert” between strands β2 and β3 (Fig. 2) (named as VR-3 using a suggested nomenclature^18^). In the eukaryotic SENP/ULP family the only other member with an insertion in the same location, albeit with a completely different sequence, corresponds to the SENP8/NEDP1 member, which deconjugates Nedd8 instead of human SUMO1/2^34,35^ (Supplementary Fig. 1). However, in the bacterial CE-clan members, an insertion in this location (VR-3) is often utilized to interact with the ubiquitin C-terminal tail (Supplementary Fig. 5). The NopD “Loop insert” (or VR-3) participates in the interaction with the C-terminal tail of both ubiquitin and SUMO, and it is essential for the NopD proteolytic activity.

### C-terminal tail comparison of SUMO2 and ubiquitin in NopD

**Fig. 3:**
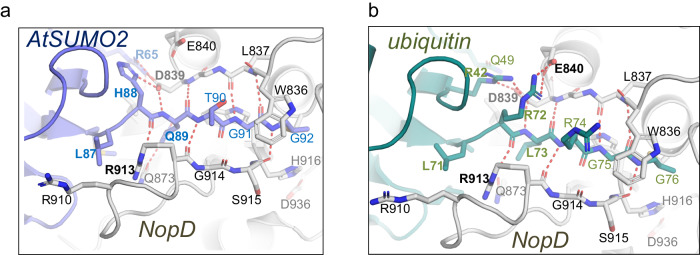
Structural details of the NopD C-terminal interaction. **a** Close-up view of the C-terminal tail of AtSUMO2 (purple) in complex with NopD (gray). **b** Close-up view of the C-terminal of ubiquitin (green) in complex with NopD (gray). Binding side-chain residues in the contact area of substrate and enzyme are shown in stick representation and labelled. Hydrogen bonds are represented by dashed red lines.

The C-terminal tails of ubiquitin and SUMO2 are buried in a NopD surface cleft that contains the active site catalytic triad, responsible for cleaving off the isopeptidic bond after the diGly motif in both SUMO and ubiquitin C-terminus. Several conserved contacts are observed despite the low-identity of the C-terminal tails of AtSUMO2 (-MLHQTGG) and ubiquitin (-VLRLRGG). Similar to other members of the eukaryotic SENP/ULP family, this interface is mainly stabilized by a large number of backbone hydrogen bonds between the C-terminal tail and NopD (Fig. 3a, b). The geometry and distances of these contacts between the C-terminal tails of AtSUMO2 and ubiquitin are relatively similar, almost complete all possible backbone bonds. Singularly, the guanidinium sidechain and carbonyl mainchain of Arg913, located in the “Loop insert” of NopD, contribute with two backbone bonds to stabilize the C-terminal tail of ubiquitin and AtSUMO2. Trp836 in NopD sandwiches the C-terminal diGly motif, as usually observed in other members of the SENP/ULP family.

All side chains residues of the AtSUMO2 and ubiquitin C-terminal tail are engaged with NopD, except Thr90 or Arg74 in either AtSUMO2 or ubiquitin, respectively (Fig. 3a, b). Gln89 in AtSUMO2 forms a hydrogen bond with Gln873, not present in the equivalent Leu73 in ubiquitin. In vitro assays with the NopD Q873N point mutant confirms the role of this hydrogen bond for AtSUMO2, and partially for ubiquitin or AtNedd8. Interestingly, whilst His88 in AtSUMO2 forms an electrostatic bond with Asp839, the equivalent Arg72 in ubiquitin binds Glu840 (Fig. 3a, b). Moreover, Leu87 and Leu71, in AtSUMO2 and ubiquitin, respectively, are located in the similar pocket formed by the “Loop insert” in NopD (Fig. 3a, b).

A previous study reported that NopD was specific for plant SUMO isoforms^25^, such as AtSUMO1 and AtSUMO2, GmSUMO (soy bean), PvSUMO (common bean), all containing a similar C-terminal sequence (-MLHQTGG), in contrast to the lack of activity for human SUMO isoforms, which contain different C-terminal sequences (-YQEQTGG in hSUMO1 or -FQQQTGG in hSUMO2/3) (Fig. 1d). The structure of the AtSUMO2-NopD complex sheds light into the SUMO ortholog specificity in plants by the contacts established by Leu87 and His88 with NopD (Fig. 3a). In particular by the electrostatic interaction of AtSUMO2 His88 with NopD Asp839, which cannot be established by the equivalent Glu or Gln in the human counterparts, and by the hydrophobic interaction of AtSUMO2 Leu87 with the “Loop insert” pocket.

### Unraveling the SUMO2 and ubiquitin interface with NopD

**Fig. 4:**
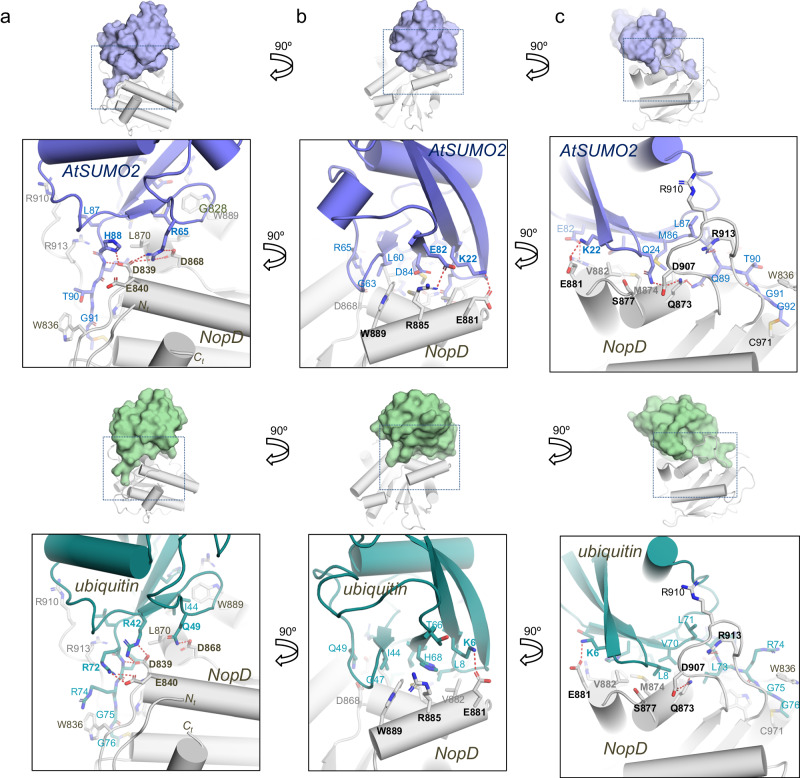
Interface between ubiquitin and AtSUMO2 globular domain with NopD. **a**–**c** Close-up view of the ribbon representation of NopD-AtSUMO2 and NopD-ubiquitin interfaces in three orthogonal views. Binding side-chain residues in the contact area are labelled and shown in stick representation. AtSUMO2 and ubiquitin residues are colored in blue and green, respectively. Hydrogen bonds and charged interactions are represented by dashed red lines.

As initially reported in the yeast ULP1-SUMO structure^36^, an extended interface between the globular domain of SUMO and ubiquitin with NopD is observed. To analyze their specific contacts, both interfaces have been divided into three orthogonal views (Fig. 4).

**Fig. 5:**
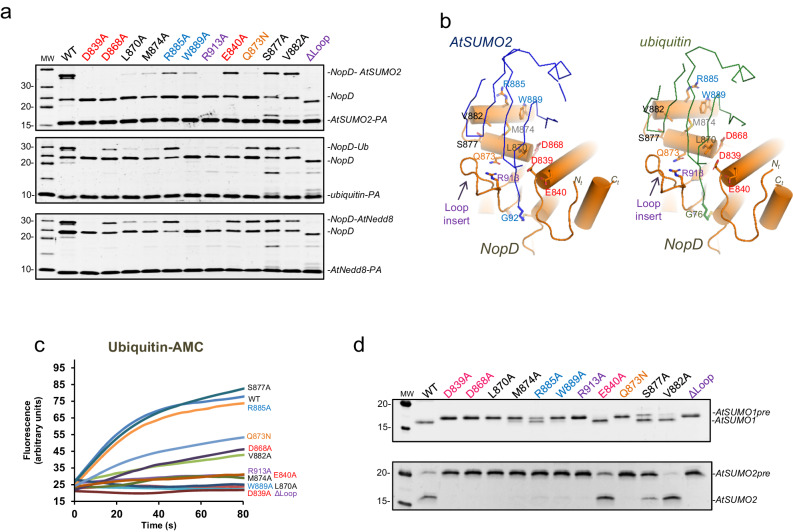
In vitro catalytic analysis of the NopD SUMO/ubiquitin specificity. **a** SDS-PAGE binding analysis of NopD wild-type and point mutants with AtSUMO2-PA (above), ubiquitin-PA (middle) and AtSUMO2-PA (below) activity-based propargylamine-derived probes. Reaction assays were performed with NopD at 1 μM using the PA probes at 4 μM for 2 h. **b** Cartoon representation of the NopD-AtSUMO2 and ubiquitin complexes. AtSUMO2 (blue) and ubiquitin (green) are shown in ribbon representation and NopD (orange) in cartoon representation. Analyzed interface residues are labeled and shown in stick representation. **c** Time-course plot of the hydrolysis of the fluorescent ubiquitin-AMC substrate with NopD wild type and point mutants. Reactions were conducted in triplicate and the average curve is displayed. **d** SDS-PAGE of end point activity assays for NopD (200 nM) using the substrates precursor of *Arabidopsis* SUMO1 and SUMO2 (1 μM).

In the first view AtSUMO2 and ubiquitin are engaged to an acidic NopD patch composed by Asp839, Glu840, and Asp868 (Fig. 4a). In the AtSUMO2 complex, Asp839 and Asp868 form electrostatic interactions with His88 and Arg65, respectively, whereas in ubiquitin they interact with Arg42 and Gln49. Glu840 only participates in the ubiquitin complex by contacting Arg72. Asp839 is highly conserved in the eukaryotic SENP/ULP family (Supplementary Fig. 1), and it is essential in the in vitro assays of SUMO, ubiquitin, and Nedd8 (Fig. 5). However, Glu840 and Asp868 display opposite results in assays with SUMO, ubiquitin and Nedd8. The electrostatic interaction of Glu840 with Arg72 is essential in ubiquitin, but not in AtSUMO or AtNedd8; whereas the Asp868 electrostatic interaction with Arg65 is essential in AtSUMO, but not for ubiquitin or AtNedd8 (Fig. 5).

In the second interface view, orthogonal to the C-terminus (Fig. 4b), the major contacts emerge from helix α4 are composed by Trp889, Arg885, and Glu881. In ubiquitin, Trp889 is located in a pocket formed by Ile44, Gly47, and His68 (all distances around 3.5 Å); whereas in AtSUMO2, Trp889 is located in a similar pocket formed by Leu60, Gly63, and Asp84 (all distances around 3.5 Å). Mutagenesis analysis highlights the essential role of Trp889 in binding and activity assays for AtSUMO, ubiquitin and AtNedd8 (Fig. 5), which is conserved and essential in the eukaryotic SENP/ULP family (Supplementary Fig. 1). Arg885 is engaged in an electrostatic interaction with Glu82 in AtSUMO2, whereas in ubiquitin it does not interact with the equivalent His68 (Fig. 4b). In vitro assays confirm the major role of Arg885 in AtSUMO, but not in ubiquitin or AtNedd8 (Fig. 5). Finally, Glu881 is at contact distance to either Lys22 or Lys6 in AtSUMO2 or ubiquitin, respectively, but the electron density maps do not show a strong interaction.

In the third interface view, contacts are established by residues emerging from helices α3 and α4 (Fig. 4c). In AtSUMO2 Gln24 and Met86 are placed in a NopD pocket formed by Leu870, Gln873, Met874 from helix α3, Val882 from helix α4, and Asp907 from the NopD “Loop insert”, whereas in ubiquitin the equivalent Leu8 and Val70 interact with a similar NopD pocket. Disruption of this hydrophobic pocket, such as in the L870A and M874A point mutants, compromises binding and deconjugation activity of NopD in AtSUMO, ubiquitin, and AtNedd8 (Fig. 5). Ser877 forms a strong hydrogen bond with the mainchain of Gln24 or Leu8 in either SUMO or ubiquitin, respectively (around 2.7 Å distance). However, the NopD S877A point mutant does not compromise our in vitro binding and activity assays (Fig. 5).

### Dissecting the deconjugation activities in tobacco leaves infiltration assays

The deconjugating activity of NopD was shown to be essential in the induction of cell death after *Agrobacterium* infiltration in leaves of tobacco plants^25^, usually used as a model to check protein targets during plant infection. We have recapitulated these experiments using full-length and catalytic domain constructs of NopD wild-type, C971A, and E840A single point mutants. Our aim was to discern between the different deconjugation activities in NopD, namely SUMO, ubiquitin, and Nedd8, by using a single point mutant, NopD E840A, which only compromises the deubiquitinating activity (Fig. 5).

**Fig. 6:**
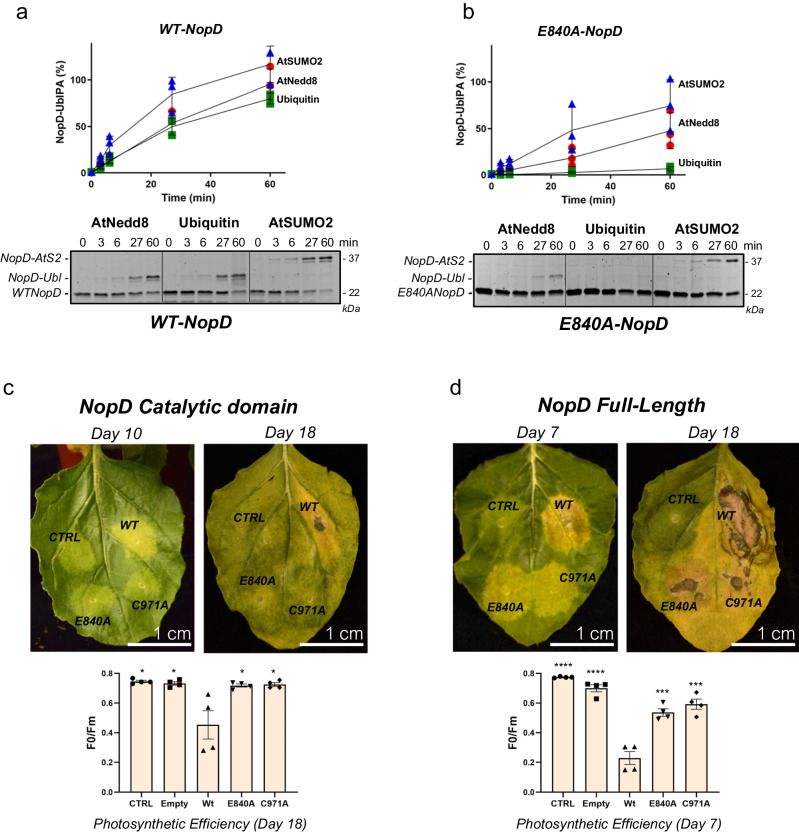
Expression of NopD and NopD mutants in plant cells induces cell death. Time-course plot of the SDS-PAGE binding analysis of NopD wild-type (**a**) and NopD E840A point mutant (**b**) with AtNedd8-PA, ubiquitin-PA and AtSUMO2-PA activity-based probes. Data values represent the mean ± SD, *n* = 3 technical replicates. Expression of the catalytic domain (**c)** or the full-length (**d**) NopD WT, NopD C971A, and NopD E840A proteins under the control of CAMV 35S promoter in different sections of a leaf was performed by infiltration of *A. Tumefaciens* transformed with pCAMBIA plasmids carrying the described NopD genes. Empty plasmid was used as a control for comparison. The photographs were taken during several days post infiltration (dpi). Cell death necrotic tissue, can be visualized in wtNopD (yellow-brown areas), whereas expression of NopD C971A or NopD E840A showed no visible effects. Quantification of cell death was measured by the reduction in photosynthetic efficiency of the tissue surrounding the injection point on each section of the leaf. Measurements around the injection point were taken for each section using four independent leaves. Therefore, data values represent the mean ± SD, *n* = 4 biological replicates. Significance was measured by a two-tailed unpaired *t* test relative to wild type. All data were analyzed with a 95% confidence interval. *P ≤ 0.05, **P ≤ 0.01, ***P ≤ 0.001, ****P ≤ 0.0001. Exact *P* values from the left to right: (**a**) 0.022, 0.027, 0.033, 0.030; (**d**) <0.0001, <0.0001, 0.0009, 0.0006.

Glu840 is a unique trait in NopD essential for binding to ubiquitin, as observed in the complex structure engaged to ubiquitin’s Arg72 (Fig. 3b). This interaction is not present in complex with SUMO (Fig. 3a), and cannot take place in complex with Nedd8, in which Arg72 is replaced by an alanine (Fig. 1d). A time-course in vitro binding analysis with activity-based probes of AtSUMO2-PA, AtNedd8-PA, and ubiquitin-PA confirmed that NopD E840A point mutant is able to discriminate between UbLs, being highly ineffective in the deubiquitinating and partially for deNeddylation activities (Fig. 6a, b).

Infiltration in tobacco leaves with the NopD catalytic domains reveals an equivalent loss of cell death phenotypes and photosynthetic efficiency for the C971A and E840A point mutants compared to the strong cell death phenotype observed in the NopD wild-type (Fig. 6c). These results indicate that cell death might be caused by the NopD deubiquitinating activity, as the loss of overall deconjugation (NopD C971A) and deubiquitination (NopD E840A) yielded comparable outcomes. Similar phenotypes, albeit within a shorter time frame, are observed with the NopD full length, which includes the long unstructured N-terminal extension, in this instance the catalytically inactivated and the E840A NopD still appears to retain some residual ability to induce cell death (Fig. 6d). Our results indicate that the cell death phenotype in tobacco leaves depends on the deubiquitinating activity of NopD. Because we cannot fully dissociate NopD deSUMOylation activity from its deubiquitinating activity by mutation, it remains possible that deSUMOylation (or deNeddylation) activity is also involved. While distant from nodulation, cell death infiltration assays might represent a useful tool to study the particular activities of UbLs in plant infection or nodulation by pathogenic or symbiotic bacteria, respectively.

## Discussion

The eukaryotic CE protease clan in humans, the SENP/ULP family, comprises six SUMO-specific and one Nedd8-specific members. Within humans, the conserved catalytic domain has evolved to discriminate between SUMO isoforms, and in, the case of SENP8/NEDP1, it has even adapted to interact with a distinct UbL modifier, Nedd8 (but not ubiquitin). Infectious bacteria, on the other hand, employ secreted effector proteins containing CE proteases to disrupt host response processes. However, in bacteria, the CE protease clan has evolved to acquire deubiquitinating activity^18^, or even unusual acetyltransferase activities, such as in the *Yersinia pestis* YopJ^37^. Thus, it appears that in different cellular contexts, the conserved catalytic fold of the CE protease family has evolved to cleave off different types of UbL modifiers.

The particular ability in the CE proteases of pathogenic bacteria to break down host-cell ubiquitin conjugates is achieved by inserting unique sequences between the conserved secondary structure elements of the catalytic domain^18^. In eukaryotic SENP/ULP members, sequence insertions are also present to distinguish SUMO isoforms, such as in SENP6 and SENP7^38^, or even in SENP8/NEDP1 to cleave off Nedd8 conjugates^34,35^. Typically, the CE proteases of pathogenic bacteria show a preference for targeting K63-linkage ubiquitin chains, which are involved in inflammatory signaling cascades in humans. In contrast, the plant pathogen XopD, from *Xanthomonas campestris*, prefers the K48-linkage ubiquitin chains, highlighting the versatility of the CE clan to modulate cleavage of different types of ubiquitin chains.

Here we have discovered that NopD, an effector protease from *Bradyrhizobium* involved in symbiotic plant nodulation, possesses multiple deconjugation activities, including deSUMOylation (*Arabidopsis* SUMO1 and SUMO2), deubiquitination (preferring cleavage of K48-linkage ubiquitin chains), and deNeddylation (human and *Arabidopsis* Nedd8). Our structural studies uncover that in contrast to *Xanthomonas* XopD, NopD shares the same binding interface for ubiquitin as it does for SUMO. This feature is particularly notable considering the substantial differences between SUMO and ubiquitin surfaces (Supplementary Fig. 4). Given the close relation between Nedd8 and ubiquitin (58% sequence identity), we anticipated a similar binding interface with NopD, as supported by our Alphafold model of the NopD-Nedd8 complex (Supplementary Fig. 7).

The contrasting modes of ubiquitin binding to NopD, compared to other members of the CE-proteases clan of pathogenic bacteria such as XopD, SdeA, ChlaDUB and OtDUB, provide a compelling illustration of convergent evolution in distant organisms. In each instance, a unique mechanism for interacting with ubiquitin is showcased. In *Xanthomonas* XopD relies on an acquired N-terminal extension to create a distinct ubiquitin interface (see Supplementary Fig. 5), whereas in *Chlamidia* ChlaDUB, *Legionella* SdeA and *Rhizobia* NopD, different sequences are inserted in the connecting VR-3 loop region to interact to ubiquitin, employing various strategies (see Supplementary Fig. 6). In *Orientia* OtDUB, instead of an insertion in the VR-3 region, a large helical domain at the C-terminus contributes to interacting with ubiquitin. In all these distant organisms a particular ubiquitin binding interface is present, displaying different orientations of ubiquitin in relation to the fixed C-terminal tail at the catalytic pocket of the protease (see Supplementary Fig. 6).

One particular feature that enables NopD to exhibit multiple deconjugation activities is the presence of an insertion, termed the “Loop insert” or VR-3 loop region, which plays a crucial role in anchoring the C-terminal tails of both AtSUMO and ubiquitin. Our in vitro analyses highlight the significance of Arg913 within this “Loop insert,” which is conserved among NopD orthologs (Supplementary Fig. 8), as it establishes similar backbone hydrogen bonds with the C-termini of both SUMO and ubiquitin.

Intriguingly, despite their low homology, Arabidopsis SUMO1 and SUMO2 share certain structural similarities with ubiquitin. Specifically, Leu87 and His88 within the C-termini of Arabidopsis SUMO1 and SUMO2 align structurally with Leu71 and Arg72 of ubiquitin. While Leu87 (AtSUMO1) and Leu71 (ubiquitin) bind a similar patch of the “Loop insert,” the positive charges of His88 (AtSUMO) or Arg72 (ubiquitin) engage either Asp839 or Glu840, respectively (Fig. 3). In contrast, human SUMO1 and SUMO2, which possess either Gln or Glu/Gln at these positions (Fig. 1d), fail to bind NopD, as initially described for Xanthomonas XopD^26^.

Another interesting feature in the NopD-ubiquitin structure is the presence of a strong well-oriented electrostatic interaction between ubiquitin’s C-terminal Arg72 and NopD Glu840 (Fig. 3 and Supplementary Fig. 5). In certain DUBs, such as USPs or OTU families^35,39,40^, the ubiquitin C-terminal Arg72 is employed as a major binding point to correctly position the ubiquitin C-terminal tail within the protease active site. Notably, our functional analysis indicated that the NopD E840A point mutant is only relevant for ubiquitin, in contrast to AtSUMO2 or AtNedd8 (Figs. 5, 6). We propose that Glu840 likely represents an evolutionary adaptation in symbiotic *Rhizobium* and *Bradyrhizobium* to enhance NopD’s deconjugation of ubiquitin.

The cross-reactivity of *rhizobia* NopD for SUMO, ubiquitin, and Nedd8 is an atypical feature within the CE protease clan. Only some other human pathogen effectors, such as ChlaDUB1, RickCE, and SdeA, has been reported to exhibit cross-reactivity for ubiquitin and Nedd8^16,18^, which is not particularly surprising given the 58% sequence identity and nearly identical C-terminal tails of ubiquitin and Nedd8 (Fig. 1d). But the multiple deconjugation activity of NopD for SUMO, ubiquitin and Nedd8 is somehow unexpected, basically by the dissimilar C-terminal tail and non-conserved surface residues. So, it seems that *rhizobia* NopD has evolved to gain deconjugation activities for SUMO, ubiquitin, and Nedd8, in a single catalytic domain by the acquisition of a “Loop insert” sequence and the maintenance of key “hot spots” in the interface that are essential for the proteolytic activity of each UbL. Considering the critical equilibrium between plant defense and symbiosis establishment, these multiple functions in a single effector, in terms of specificity and activity, could constitute an advantage to reach the required equilibrium. This discovery not only unveils the fascinating evolutionary adaptations of these proteins but also underscores the significance of NopD in expanding our understanding of ubiquitin and UbL interactions.

## Materials and methods

### Plasmids, Cloning and Point Mutation

The synthesized gene encoding the catalytic domain of NopD cloned into the pMx vector was purchased from Thermo Fisher Scientific. All constructs of pET28a-NopD were amplified by PCR and ligated using the “Restriction Enzyme Free PCR” methodology^41^. The NopD point mutants constructs were generated by different primers and were created by the QuickChange site-directed mutagenesis kit (Stratagene). All primers are shown in the Supplementary Table 1. The plasmid of pCAMBIA-NopD Full-length and catalytic domain were cloned from pET28-NopD Full-length (a gift from Dr. Christian Staehelin). *Arabidopsis thaliana* SUMO1 and SUMO2 precursors were cloned into pET28a in Dr. L. M. Lois lab. All plasmids for the pTXB1-HsSUMO2G, pTXB1-AtSUMO2G, pTXB1-UbiquitinG, and pTXB1-AtNedd8G expression were cloned using the “Restriction Enzyme Free PCR” methodology^41^.

### Protein expression and purification

The NopD-CD (catalytic domains), human SUMO2, *A. thaliana* SUMO2 (AtSUMO2), and human ubiquitin, as well as *A. thaliana* Nedd8 expression constructs, were transformed into E. coli Rosetta (DE3) cells (Novagen). Bacterial cultures were grown at 37 °C to OD600 = 0.7–0.8, and IPTG was added to a final concentration of 0.5 mM. Cultures were grown an additional 5 h at 30 °C and harvested by centrifugation.

For NopD-CD and SUMO precursors, cells were suspended in a solution containing 20 mM Tris-HCl (pH 8.0), 350 mM NaCl, 10 mM imidazole, 20% sucrose, 1 mM DTT, and 0.1% IGEPAL CA-630. Subsequently, the cells were broken by sonication. After the removal of cell debris by centrifugation, proteins were purified using nickel affinity chromatography with Ni Sepharose 6 Fast Flow (GE Healthcare) and eluted with a solution composed of 20 mM Tris-HCl (pH 8.0), 350 mM NaCl, 300 mM imidazole, and 1 mM DTT. The proteins underwent further purification through gel filtration (Superdex 75; Cytiva) equilibrated with 20 mM Tris-HCl (pH 8.0), 250 mM NaCl, and 1 mM DTT. Finally, for NopD-CD, GF fractions containing the target protein were pooled, diluted to 50 mM NaCl, and applied to an anion exchange resin (Resource S; Cytiva). The elution was carried out with a 0–1 M NaCl gradient from 0 to 50% in 20 mM HEPES (pH 8.0) and 1 mM DTT. All proteins were then concentrated using an Amicon Ultra ultrafiltration device (Millipore) and snap-frozen in liquid nitrogen before storage at −80 °C.

### Preparation of the NopD complexes with AtSUMO2 and ubiquitin

The method to generate AtSUMO2-PA or Ub-PA protein derivatives has been previously described^42^. NopD-CD protein and AtSUMO2-PA/Ub-PA (1:4 ratio) were incubated for 3 h at 30 °C. After that, the buffer was changed to 20 mM HEPES 7.42, 50 mM NaCl, 1 mM DTT by rounds of concentration and dilution with Amicon Ultra-30K ultrafiltration device. Complexes were purified using an anion exchange resin (Resource S; Cytiva) as described above.

### Crystallization and data collection

NopD-AtSUMO2-PA and NopD-Ub-PA were concentrated to 12 mg/mL for crystallization screening. Crystallization experiments were performed at 18 °C by sitting drop vapor diffusion method. NopD-AtSUMO2-PA crystals grew up in a protein mixture with an equal volume of a condition solution containing 0.1 M Imidazole, pH 7.0, and 50% v/v MPD. NopD-Ub-PA crystals grew up in a protein mixture with an equal volume of a condition solution containing 0.1 M Imidazole pH 8.0 and 10% w/v PEG8000. Crystals were harvested after 1–2 weeks and soaked 5–10 s in the crystallization buffer supplemented with 15% ethylene glycol, and then snap-frozen in liquid nitrogen to storage.

Diffraction data were collected at beamline BL13-XALOC at the ALBA synchrotron (Barcelona, Spain)^43^. NopD-AtSUMO2PA and NopD-UbPA get a resolution: 1.50 Å and 1.94 Å, respectively. Resolution Data processing was conducted by AutoProcesing with MxCUBE^44,45^. Structures were solved by molecular replacement with NopD AlphaFold2 model as a search mode^46^. Following rounds of model building and refinement were carried out with Coot and Phenix^47,48^. Structures reported have been deposited in the Protein Data Bank under accession codes 8OI3 and 8RQI.

### Activity-based binding probe assays

NopD wild-type and active site mutant (C971A) were prediluted to 1 μM in reaction buffer 20 mM Tris-HCl (pH 8.0), 250 mM NaCl, and 4 mM DTT, and combined 1:4 with 4 μM of Ub-PA, AtSUMO1-PA, AtSUMO2-PA and hSUMO2-PA for 2 h at 30 °C. The reaction was stopped by adding SDS-PAGE loading buffer and checked by SDS-PAGE.

### AMC-substrate hydrolysis assays

NopD wild-type and point mutants were incubated with Nedd8-, ubiquitin-, SUMO1- or SUMO2-AMC at 30 °C and measured the fluorescence emission using 345 nm excitation and 445 nm emission wavelengths using a Jasco FP-8200 spectrofluorometer. All measurements were carried out in triplicate with 25 nM NopD and 0.1 μM UbL-AMC substrate in a buffer containing 100 mM NaCl, 20 mM Tris-HCl pH 8, 10 mM DTT.

### In vitro de-ubiquitination assays

Protease activity was measured by incubating 3 µM di-ubiquitin (K6, K11, K27, K29, K33, K48, K63) with 600 nM of purified NopD wild type at 30 °C in a buffer containing 20 mM Tris-HCl (pH 8.0), 250 mM NaCl, and 2 mM DTT. SDS-BME loading buffer was used to terminate the reactions, and gel electrophoresis was used to examine the results (PAGE). SYPRO staining was used to identify proteins (Bio-Rad). A Gel-Doc machine and integration software were used to detect the products (ImageLab; Bio-Rad).

### In vitro de-SUMOyation assays

Protease activity was measured by incubating precursor of *Arabidopsis thaliana* SUMO1/2 with purified 200 nM of NopD wild type and mutants at 30 °C in a buffer containing 20 mM Tris-HCl (pH 8.0), 250 mM NaCl, and 2 mM DTT. SDS-BME loading buffer was used to terminate the reactions after 2 h, and gel electrophoresis was used to examine the results (PAGE). SYPRO staining was used to identify proteins (Bio-Rad). A Gel-Doc machine and integration software were used to detect the products (ImageLab; Bio-Rad).

### Cell-Death Tobacco Leaves assays

*Agrobacterium tumefaciens* GV3101 cells were transformed with the constructs pCAMBIA-NopD-CD (Catalytic Domain) or pCAMBIA-NopD-FL (Full length) and grown in YEB plates supplemented with rifampicin (100 μg/ml), gentamycin (50 μg/ml) and Kanamycin (25 μg/ml). Single transformed colonies (Checked by colony PCR) were grown o/n in 3 ml YEB medium supplemented with the same antibiotics at 28 °C. Fresh medium (20 mL) was inoculated with the precultures and grown o/n at 28 °C. The cultures were centrifuged and collected cells were resuspended in induction buffer (10 mM MgCl_2_, 10 mM MES, 150 μM acetosyringone, pH=5.6) to achieve OD_600_ = 4. The mixture was incubated at room temperature for 3 h and then mixed with the same volume of a culture harboring vector pGWB702-HCProWMV at the same OD to prevent silencing^49^ and induction buffer for a final OD = 0.5 for each strain. Mixtures were infiltrated in two different leaves of 5–10 3–4-week-old *N. benthamiana* plants grown under normal greenhouse conditions as described^49^. Agroinfiltrated leaves were collected at sequential days post-infiltration (dpi) and phenotypes recorded using a Camera NIKON D7000. Cell-death was quantified as the loss of photosynthetic capacity in the infiltrated regions of the leaves by measuring the In vivo Chl fluorescence at room temperature using a pulse modulated amplitude fluorimeter (MAXI-IMAGING-PAM, Heinz Waltz GmbH, Germany). Photosynthetic parameter of variable to maximum fluorescence (*F*v/*F*m) weas measured as described elsewhere^50^. Briefly, Leaves were first dark-adapted for 30 min, and then a very short saturating pulse (SP) was applied. Fluorescence signal before (*F*o) and after (*F*m) the SP was recorded to estimate the maximum quantum yield of photosystem II (PSII) (*F*v/*F*m).

### Statistics and reproducibility

Statistical analysis was conducted using Prism 5.0 software. A two-tailed unpaired t-test was performed relative to the wild type. Statistical *p* values are presented to at least two decimal places and are typically denoted as follows: *P ≤ 0.05, **P ≤ 0.01, ***P ≤ 0.001, ****P ≤ 0.0001. The data values in Fig. 6 represent the mean ± SD and *n* = 4 biological replicates. Error bars in all graphs represent the mean ± SD.

### Supplementary information

Supplementary Information

Description of Additional Supplementary Files

Supplementary Data 1

## Data Availability

Structures reported have been deposited in the Protein Data Bank under accession codes 8OI3 and 8RQI. All other data supporting the findings of this study are available within the article and its supplementary information files. All uncropped gels are available in Supplementary Fig. 9. The source data behind the graphs in the paper can be found in Supplementary Data 1.
