# Supplementary material for: Broad-spectrum ubiquitin/ubiquitin-like deconjugation activity of the rhizobial effector NopD from Bradyrhizobium (sp. XS1150)
Source: Commun Biol. 2024 May 27;7:644. doi: 10.1038/s42003-024-06344-w (PMC11130253; doi:10.1038/s42003-024-06344-w)
Supplement: Supplementary file 1 — Supplementary Information [file 42003_2024_6344_MOESM1_ESM.pdf]

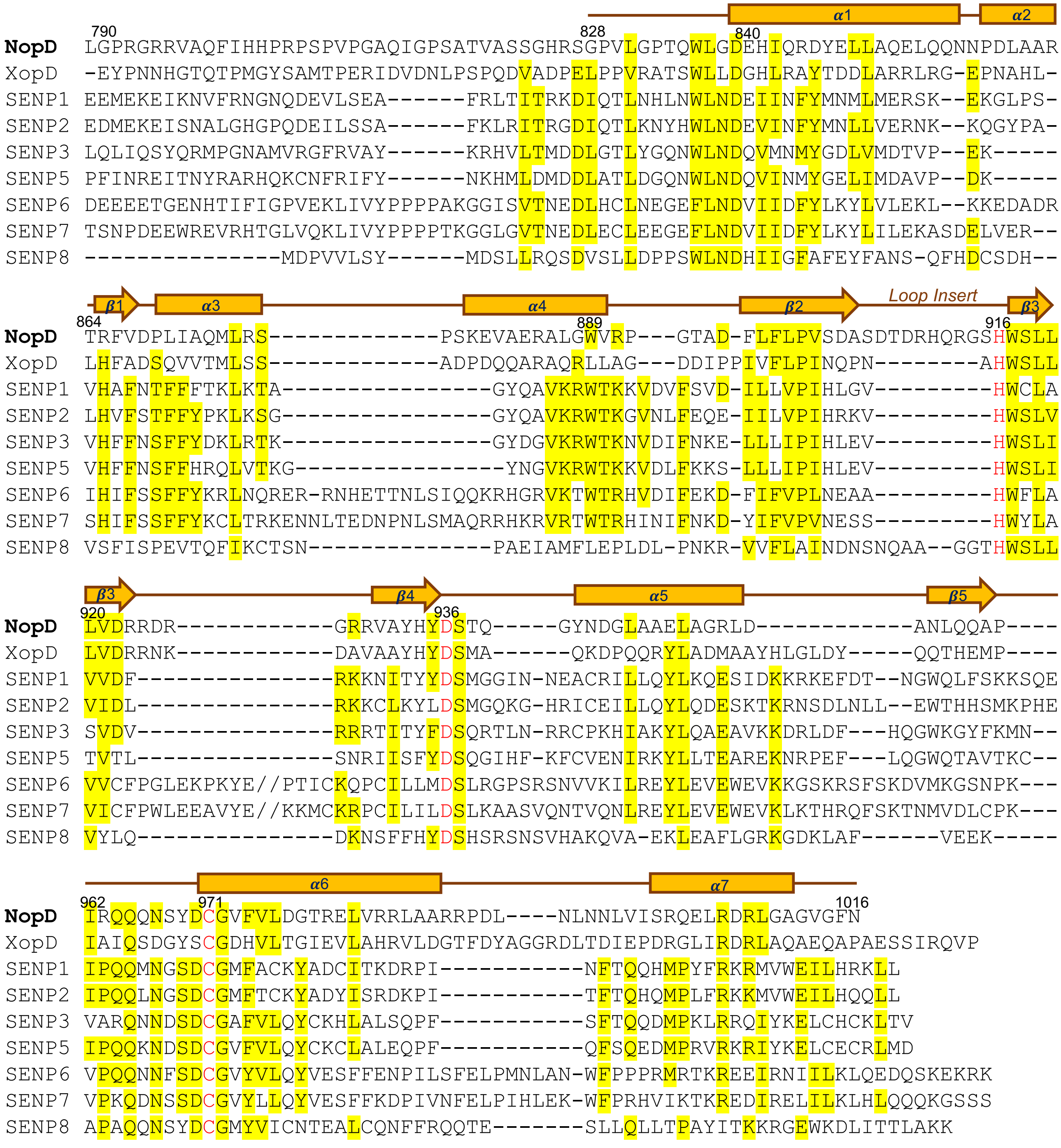

Supplementary Figure 1. Structural alignment of the *Bradyrhizobium* NopD catalytic domain with the catalytic domains of *Xanthomonas campestris* XopD, and the human SENP/ULP family members: SENP1, SENP2, SENP3, SENP5, SENP6, SENP7 and SENP8/NEDP1. A yellow backdrop indicates side chain homology with 80% conservation in the alignment. Red represents the three active site catalytic residues. NopD secondary structures (α-helix and β-strands) are numbered and labelled.

a b

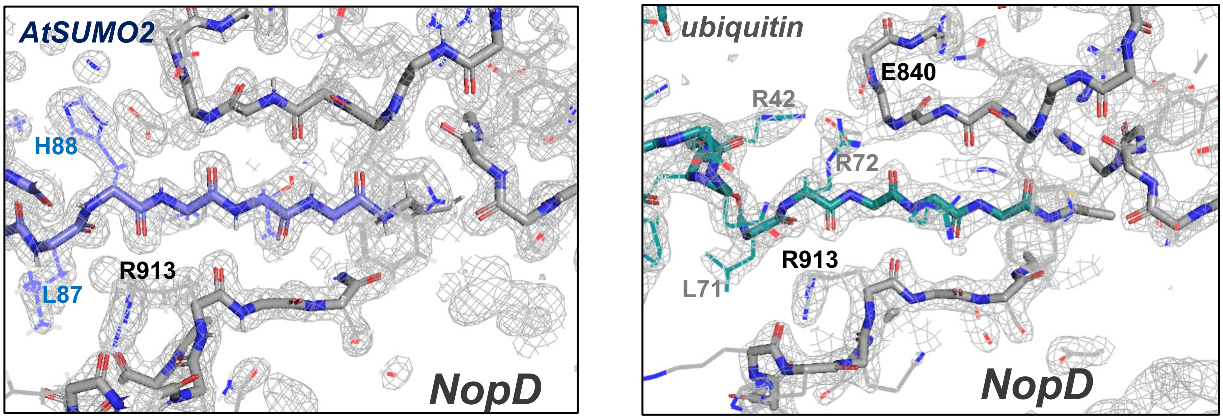

Supplementary Figure 2. Electron density maps of the C-terminal tails. Detail of the 2FoFc electron density maps around the C-terminal region of AtSUMO2/Ubiquitin in complex with NopD (grey), contoured at 2σ. Backbone chain is shown in stick representation, whereas side chains are represented in line. Some binding residues are labelled.

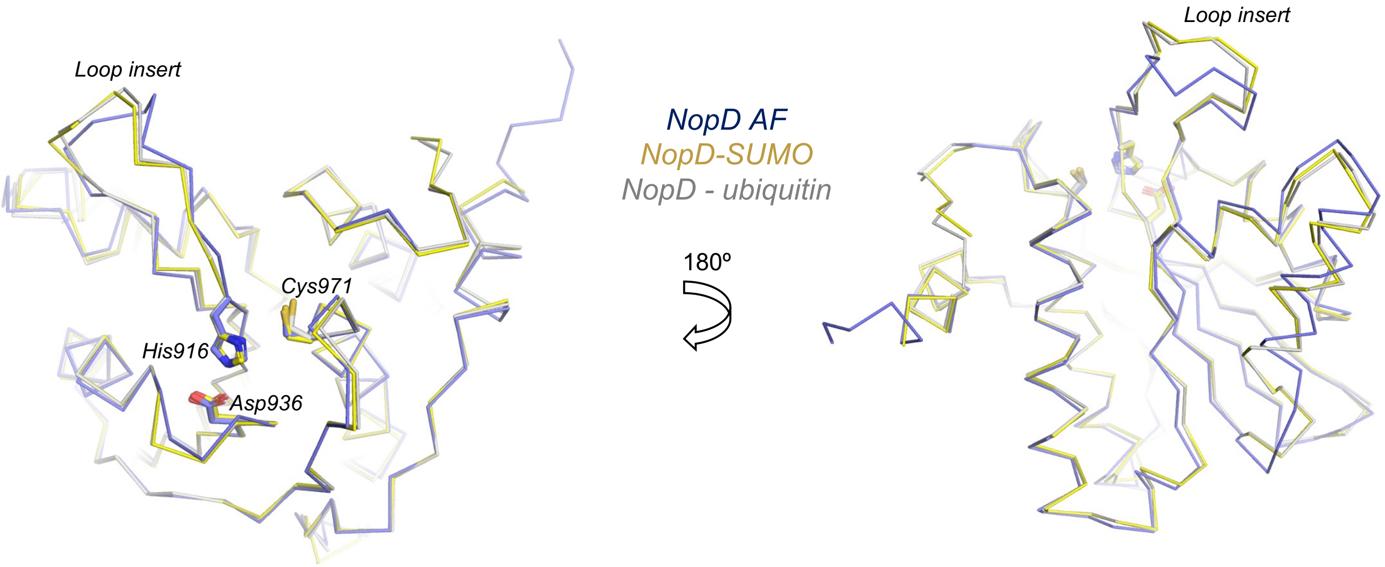

Supplementary Figure 3. Structural alignment of the catalytic triad residues of NopD in the complex structures**.** Two views of the overlapping ribbon structure of the unbound NopD AlphaFold-2 model (blue), NopD-AtSUMO2 complex (yellow) and NopD-ubiquitin complex (grey). SUMO2 and ubiquitin has been removed from the picture. Location of the “Loop insert” is marked. Catalytic residues are labeled and shown in stick representation.

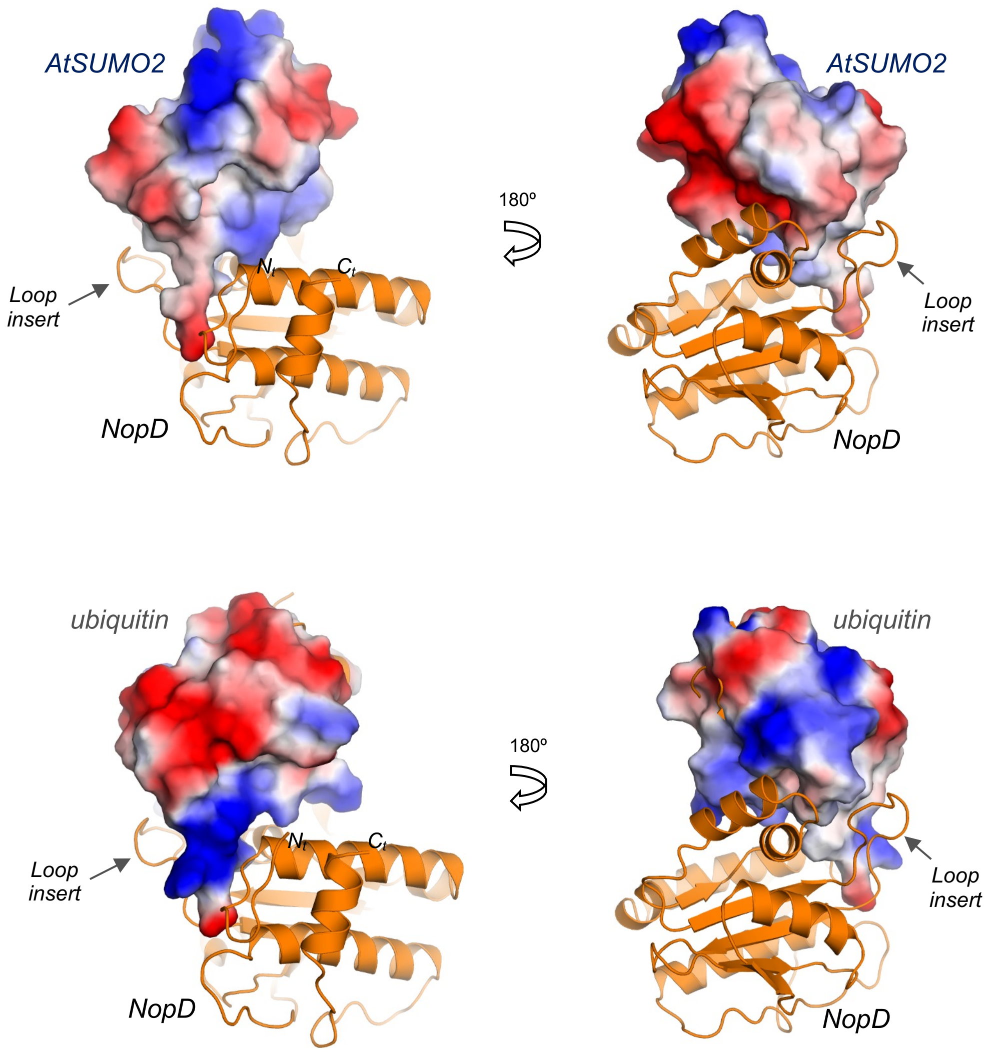

Supplementary Figure 4. Electrostatic potential surface representation of AtSUMO2 and ubiquitin in complex with NopD. Two views of electrostatic potential surface representation for AtSUMO2 and ubiquitin in complex with NopD. Red and blue colours indicate negative and positive charges, respectively. NopD is shown in an orange cartoon representation. N-terminal end, C-terminal end and Loop insert are marked and labelled.

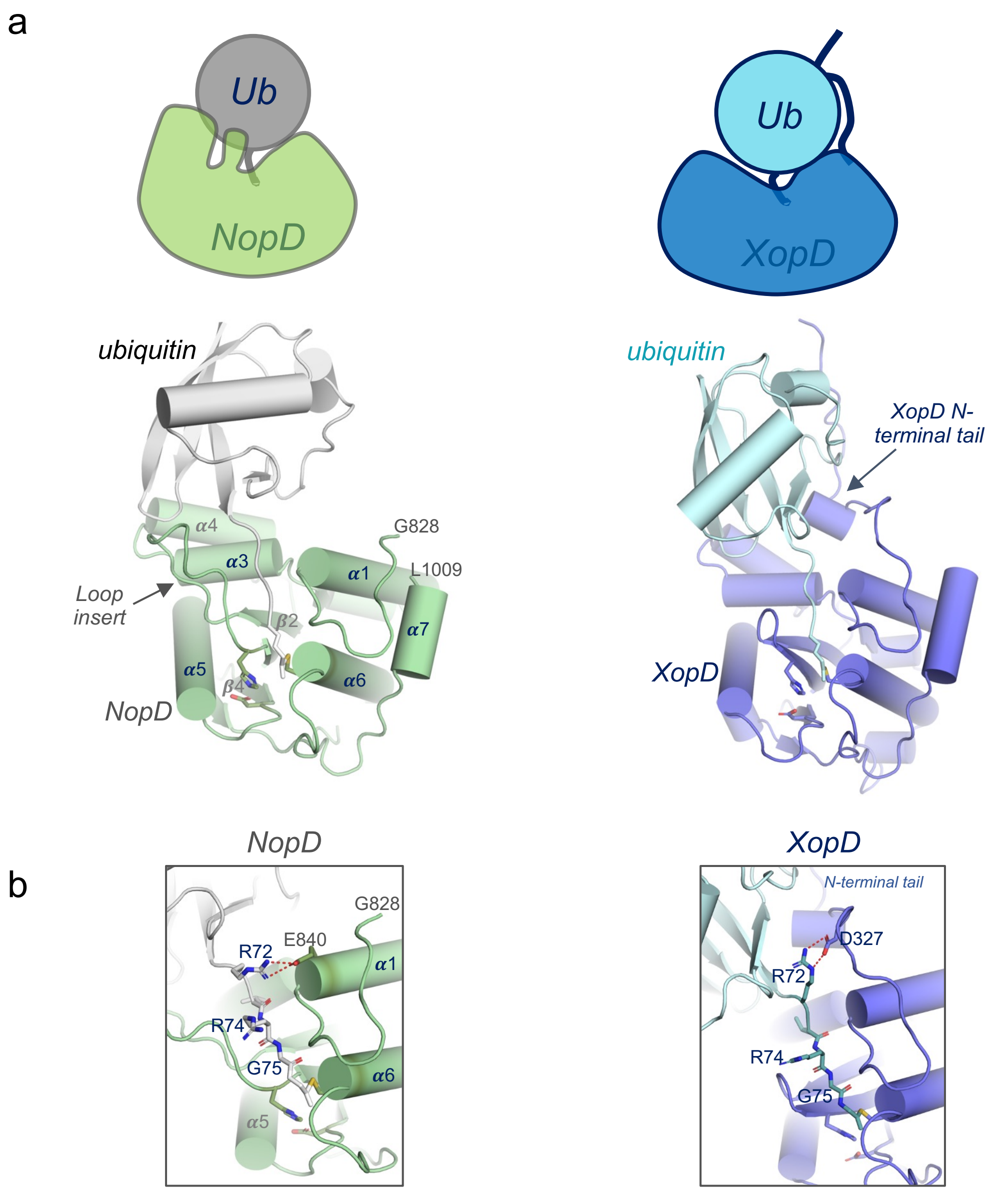

Supplementary Figure 5. a, Cartoon representation of the NopD-Ubiquitin (green) and *Xanthomonas campestris* XopD-Ubiquitin (blue) (PDB: 5JP3) complexes. The catalytic residues are shown in stick representation. Loop insert in NopD is labeled and marked. XopD N-terminal tail extension is indicated**. b**, Close-up views of the C-terminal tails of ubiquitin in NopD-ubiquitin complex (green) and in the XopD-ubiquitin complex (purple). Relevant residues are labeled and shown in stick representation. Ubiquitin Arg72 electrostatic interactions are represented by dashed red lines.

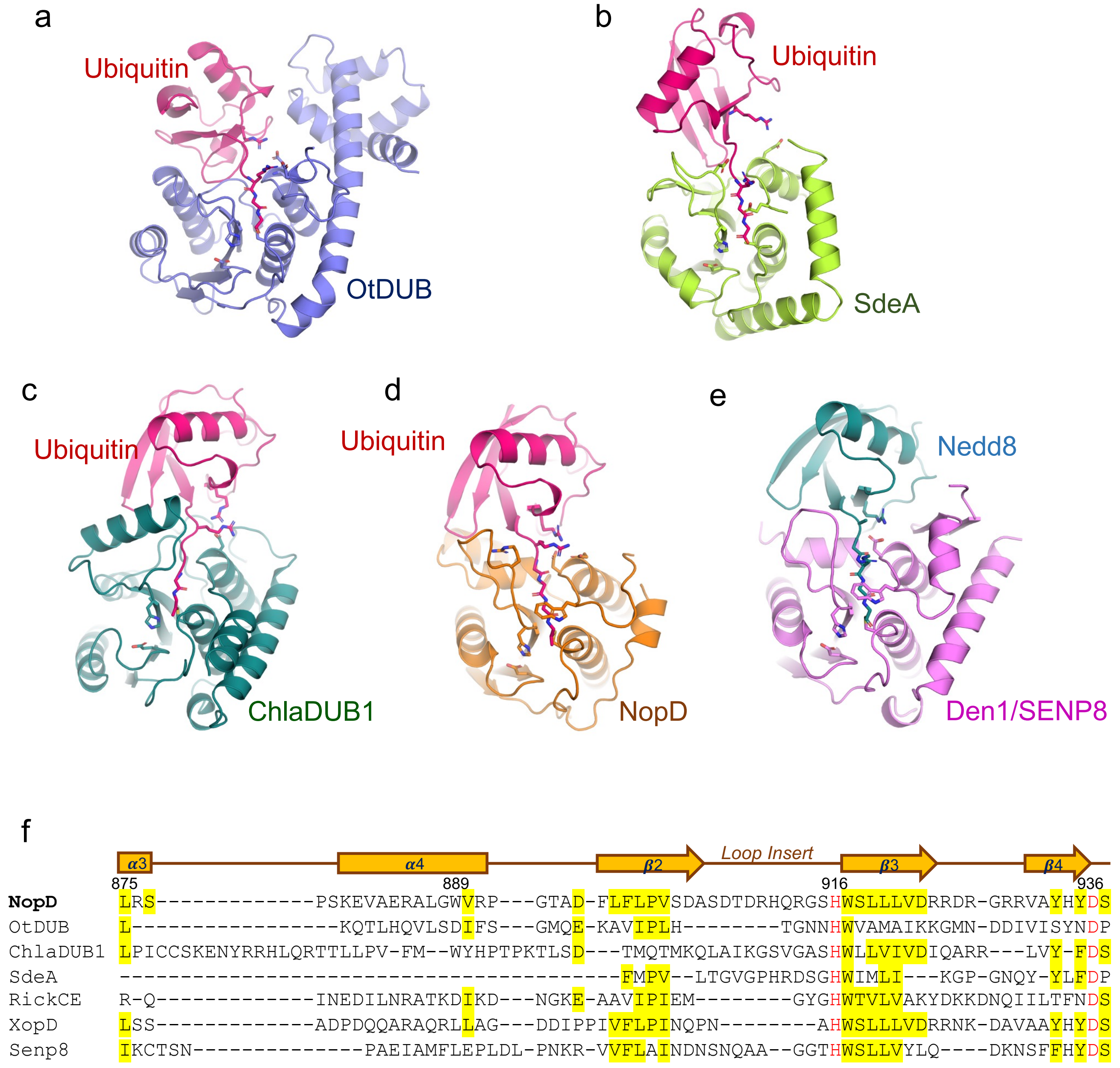

Supplementary Figure 6. Cartoon representation of **a**, the OtDUB-Ubiquitin (6UPU); **b**, SdeA-Ubiquitin (6WTG); **c**, ChlaDUB1-Ubiquitin (6GZS); **d**, NopD-Ubiquitin; and **e**, Den1/SENP8-Nedd8 (1XT9). The catalytic residues are shown in stick representation**. f**, Structural alignment of the *Bradyrhizobium* NopD catalytic domain with the catalytic domains of *Orientia tsutsugamushi* OtDUB, *Chlamydia trachomatis ChlaDUB1, Legionella pneumophila* SdeA, *Rickettsia bellii* RickCE, *Xanthomonas campestris* XopD, and the human SENP8/NEDP1. A yellow backdrop indicates side chain homology with 80% conservation in the alignment. Red represents the active site catalytic residues. NopD secondary structures (α-helix and β-strands) are numbered and labelled.

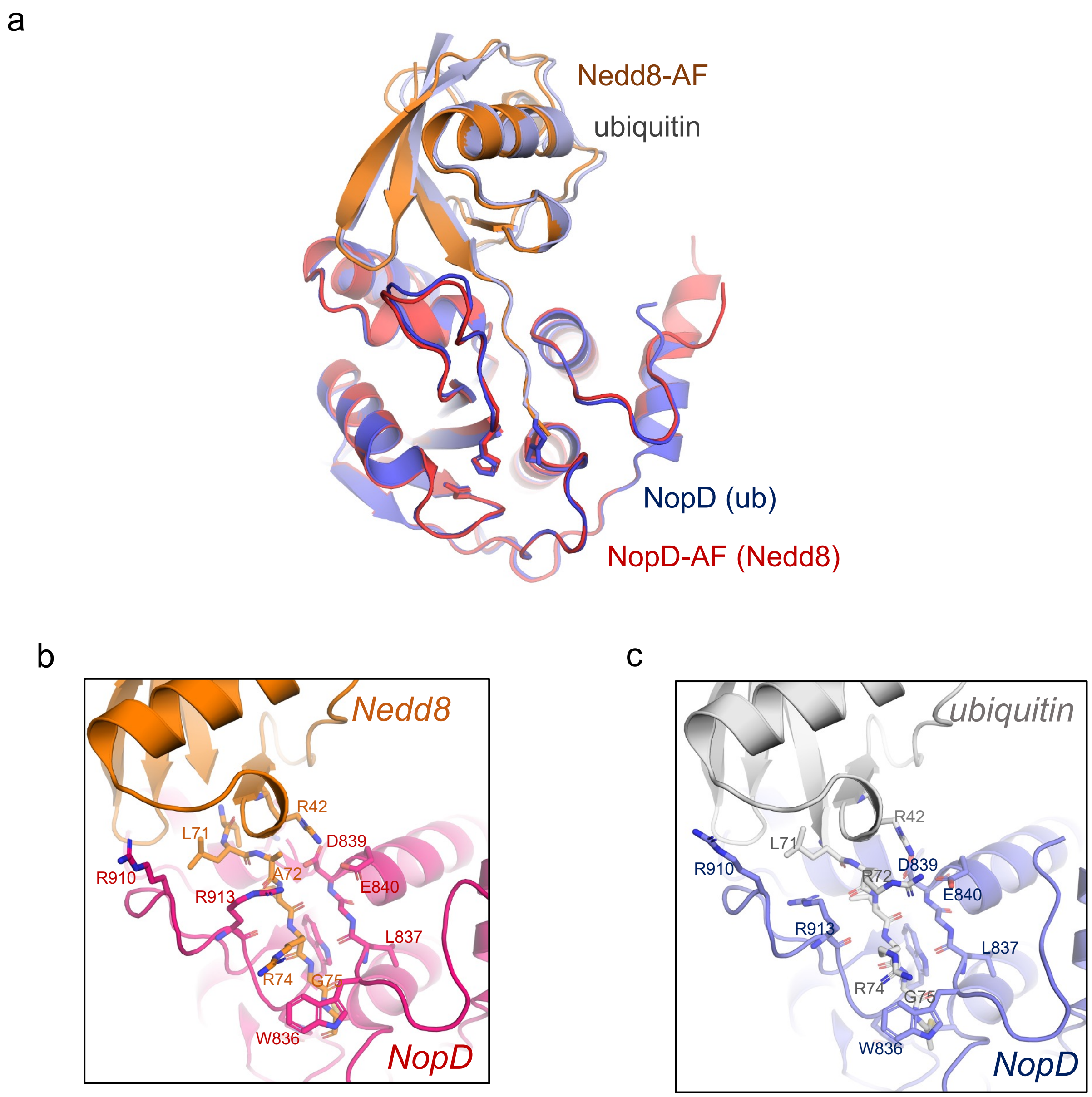

Supplementary Figure 7. a, Cartoon representation of the overlapping of the NopD-Ubiquitin complex with the Alphafold model of NopD-Nedd8 complex. The catalytic residues are shown in stick representation**. b-c**, Close-up views of the C-terminal tails of ubiquitin in the NopD-ubiquitin complex (**b**) and of Nedd8 in the Alphafold model of NopD-Nedd8 complex (**c**). Relevant residues are labeled and shown in stick representation.

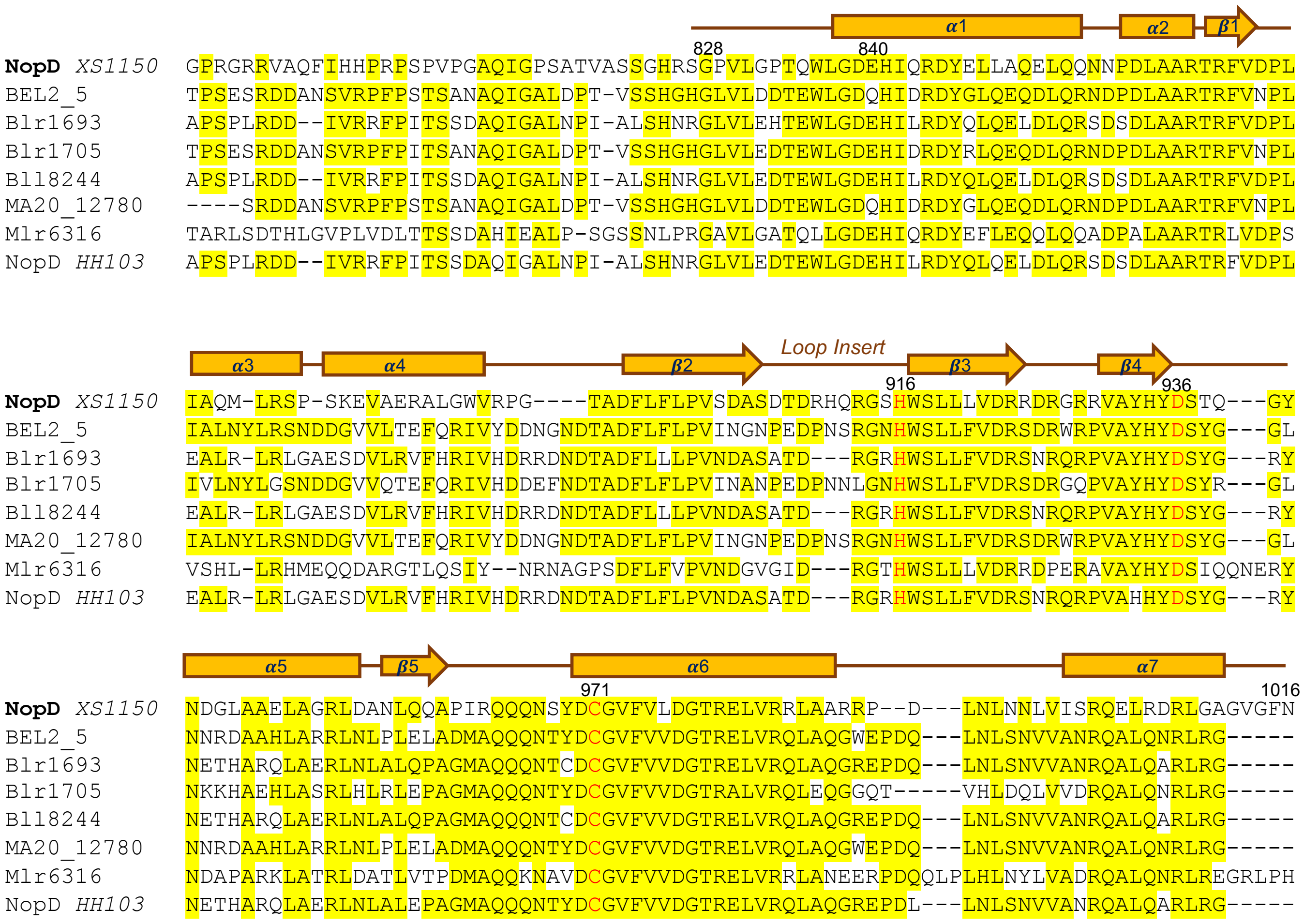

Supplementary Figure 8. Sequence alignment of the conserved catalytic domains of NopD-related proteins. Catalytic triad residues are marked in red. A yellow backdrop indicates 80% identity in the alignment. NopD secondary structures (α-helix and β-strands) are numbered and labelled in orange. NopD XS1150 is from *Bradyrhizobium* sp. XS1150, GenBank: MF100854.1; BEL2_5 is form *Bradyrhizobium elkanii* USDA 61, GenBank: AKS25901.1; Blr1693 is from *Bradyrhizobium diazoefficiens* USDA 110, GenBank: BAC46958.1; Blr1705 is from *Bradyrhizobium diazoefficiens* USDA 110, GenBank: BAC46970.1; Bll8244 is from *Bradyrhizobium diazo efficiens* USDA 110, GenBank: BAC53509.1; MA20_12780 is from *Bradyrhizobium japonicum*, GenBank: KGT79298.1; Mlr6316 is from *Mesorhizobium japonicum* MAFF 303099, GenBank: BAB52630.1; NopD HH103 is from *Sinorhizobium fredii* HH103, GenBank: CCE98838.1.

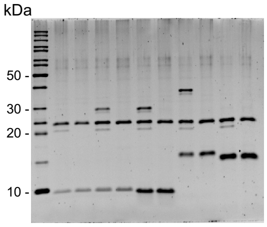

Fig. 1a

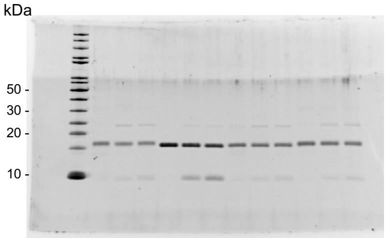

Fig. 1b

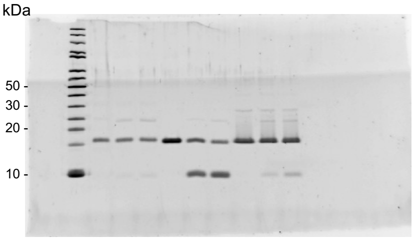

Fig. 1b

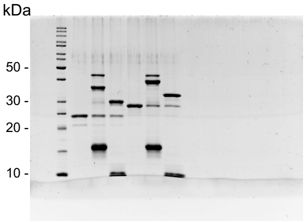

Fig. 2f

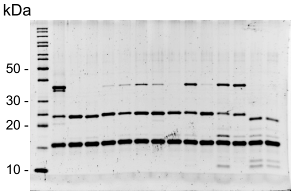

Fig. 5a

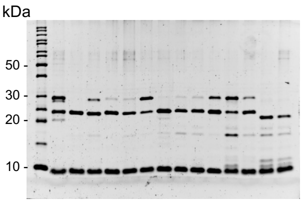

Fig. 5a

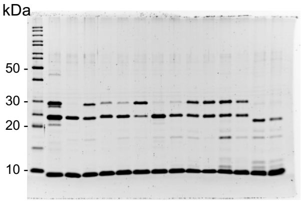

Fig. 5a

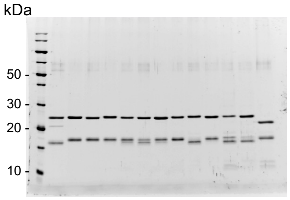

Fig. 5d

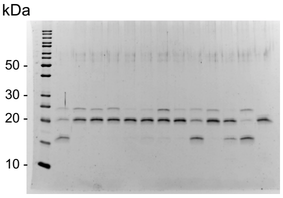

Fig. 5d

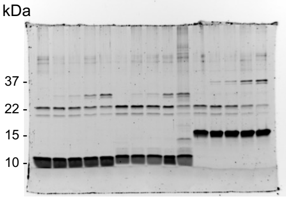

Fig. 6a

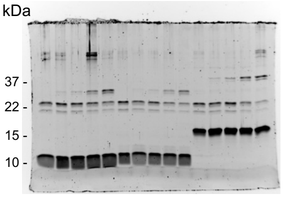

Fig. 6a

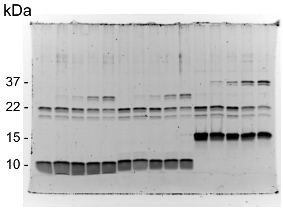

Fig. 6a

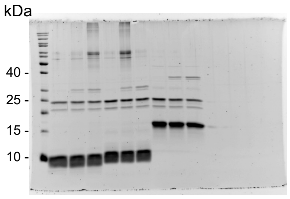

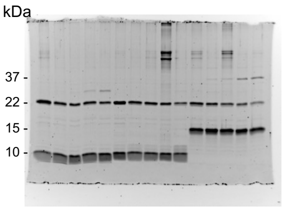

Fig. 6b

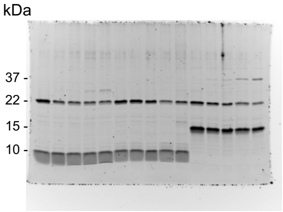

Fig. 6b

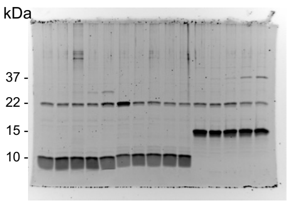

Fig. 6b

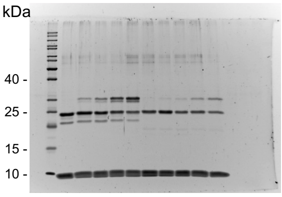

Additional gels
Fig. 6a,b

Unedited gel images

NopD 833 fw	CTGGTGCCGCGCGGCAGCCATGCGACACAGTGGCTGGGTGATGAACATATT
NopD 833 rv	GTCGACGGAGCTCGAATTCGGATCCTTAGTTAAAACCAACACCGGCACCT
NopD 827 fw	CAGGATACAGTCTAACCAAGCGAGAGGATAAGCATTTTTCC
NopD 791 fw	TGGTGCCGCGCGGCAGCCATGGTCCTCGTGGTCGTCGTGTT
NopD 798 fw	CTGGTGCCGCGCGGCAGCCATGCACAGTTTATTCATCATC
PTXB1-AtSUMO2Gfw	ACTTTAAGAAGGAGATATACATATGTCTGCTACTCCGGAAGAAGACAAG
PTXB1-AtSUMO2G rv	AGTGCATCTCCCGTGATGCAACCAGTCTGATGAAGCATTGCAT
PTXB1-AtNEDD8Gfw	TAAGAAGGAGATATACATATGATCAAGGTTAAAACCCTGACCGGC
PTXB1-AtNEDD8G rv	ACTAGTGCATCTCCCGTGATGCAACCACGCAGTGCCAGAACCA
∆ A108-G117 Fw kpnI	GATTTTCTGTTTCTGCCGGTTAGTGATGGTACC
∆ A108-G117 Rv kpnI	GATCAACCAGCAGCAGGCTCCAATGGCTGGTACC
NopD D42A fw	GTCCGACACAGTGGCTGGGTGCTGAACATATTCAGCGTGA
NopD D42A rv	TCACGCTGAATATGTTCAGCACCCAGCCACTGTGTCGGAC
NopD D71A fw	GCACGTACCCGTTTTGTTGCTCCGCTGATTGCACAGATGCTGCGTA
NopD D71A rv	TACGCAGCATCTGTGCAATCAGCGGAGCAACAAAACGGGTACGTGC
NopD Leu73A fw	GTACCCGTTTTGTTGATCCGGCGATTGCACAGATGCTGCGTA
NopD Leu73A rv	TACGCAGCATCTGTGCAATCGCCGGATCAACAAAACGGGTAC
NopD M77A fw	GTTGATCCGCTGATTGCACAGGCGCTGCGTAGCCCGAGCAA
NopD M77A rv	TTGCTCGGGCTACGCAGCGCCTGTGCAATCAGCGGATCAAC
NopD R88A fw	GAGCAAAGAAGTTGCAGAAGCCGCATTAGGTTGGGTTCGTCCGGGTA
NopD R88A rv	TACCCGGACGAACCCAACCTAATGCGGCTTCTGCAACTTCTTTGCTC
NopD W92A fw	GTTGCAGAACGCGCATTAGGTGCGGTTCGTCCGGGTACAGCAGA
NopD W92A rv	TCTGCTGTACCCGGACGAACCGCACCTAATGCGCGTTCTGCAAC
NopD R116A fw	GCGATACCGATCGTCATCAGGCTGGTAGCCATTGGAGCCT
NopD R116A rv	AGGCTCCAATGGCTACCAGCCTGATGACGATCGGTATCGC

Supplementary Table 1. Primers used in this work.
